# Investigation of the Impact of Extraneous Odours on the Detection Capability of Explosive Detection Dogs Under a Controlled Test Environment

**DOI:** 10.3390/ani16040656

**Published:** 2026-02-19

**Authors:** Christopher Becher, Michaela Schneider, Stephan Maurer, Savanna Sewell, Jörg Schulenburg, Peter Kaul

**Affiliations:** 1 Institute for Safety and Security Research, Hochschule Bonn-Rhein-Sieg, 53359 Rheinbach, Germany; 2 Bundeswehr School of Dog Handling, Gräfin-von-Maltzan-Kaserne, 56766 Ulmen, Germany; m.schneider@verhaltenundtierschutz.de (M.S.); joergschulenburg@bundeswehr.org (J.S.)

**Keywords:** canines, detection, explosives, odour, TNT, masking, behaviour, detection dogs, odour perception

## Abstract

In this work, an experimental procedure that enables testing of canine detection capabilities is described. The developed testbed includes an experimental setup with six exchangeable detection/sniffing boxes for odour masking experiments as well as an air-conditioned (adjustable temperature and humidity) test environment. This design is used to test the masking effects of petroleum and n-decane in high and low concentrations on the detection probability of targets containing technical-grade TNT on explosive detection dogs. The potential influence of the masking agents at different concentrations was investigated with eight canines and, in total, more than 1250 test runs. Within the limits of this investigation, no negative impact of the masking agent on the canine detection capabilities (probability of the successful detection of the target) could be found.

## Simple Summary

This study describes an experiment designed to test how well dogs can detect explosives when exposed to substances that might mask the scent. It used six interchangeable sniffing boxes to test how the masking agents affected the dogs’ ability to detect TNT (a common explosive). The study involved eight trained detection dogs and over 1150 test runs. The main goal was to see if the masking agents at high and low concentrations would make it harder for the dogs to successfully detect TNT. The results showed that, under the conditions tested, none of the masking agents used had a significant negative effect on the dogs’ detection abilities. This experiment is important for understanding how environmental factors, like odour masking, might impact canine detection in real-world situations, such as security screening or military operations.

## 1. Introduction

Dogs are—perhaps with the exception of some brachycephalic breeds [1]—distinctly macrosmatic mammals. When compared to humans, dogs have a larger surface area of the olfactory epithelium, significantly more olfactory receptor neurons and more functional odour receptor genes (≈800 functional genes vs. ≈400) [2,3,4]. In addition, when sniffing, inspiratory and expiratory flows decouple via lateral nasal slits and continuously draw fresh air over the nasal epithelium [1,2,5]. Dogs achieve very low detection limits for certain target substances down to the ppt range, depending on the odorant and training, and can separate complex mixtures [6,7,8,9]. They combine capabilities of extraordinary odour perception (sensitivity and specificity), high mobility and the ability to rapidly search and autonomously follow an odour plume to its source. Dogs are also able to learn new target odours in a relatively short time, which remains currently unmatched by technical detection systems [10].

They are widely used for many different detection tasks such as the detection of explosives, drugs and human remains [11,12,13], mantrailing [14,15], and detection of invasive or protected species [10,16,17]. There is also interest in using the olfactory abilities of dogs for medical detection purposes [7,18,19,20].

Explosive detection is a challenging task due to the fact that vapour pressure, evaporation rates and odour concentrations are very small for most energetic materials [21]. Nevertheless, it could be shown that canines are able to detect surface contaminations of small, point-like odour sources [22] and are therefore used as one of the methods for the detection of explosives used in improvised explosive devices (IEDs).

Despite an increasing amount of research being undertaken concerning the limitations of the abilities of scent dogs, no clear-cut answers to many phenomena such as the potential impact of odour masking can be found in the literature. The masking of odours by more volatile substances cannot be ruled out when detecting explosives, especially when taking their generally low vapour pressure into account.

It has been shown [23,24] that the masking of odours for human perception is possible through specific masking substances. Huang et al. [25] demonstrated that the application of sub-threshold masking odours might be more efficient when mixtures of masking odours instead of single substances are applied. Burseg et al. [23] also found that the masking substance used was not effective in masking a different but similar target substance, showing that masking can be very substance specific. However, substance-specific masking is not the only form of masking. Masking can also refer to the prevention of target emissions in the air (for example, by absorption) [26], as well as the addition of a further substance or mixture in order to change the perceived odour [27,28,29].

When Laing et al. [30] used trained rats to detect a single substance that was masked by another single substance, the rats’ correct response rapidly decreased in the presence of a high concentration of the masking substance. A publication from L Waggoner showed a different masking effect between a mixture and a pure substance and also that the performance of the dogs decreased with increasing concentrations of a complex masking mixture, while a pure masking substance only affected the performance of the dogs at very high concentrations [31].

While humans struggle to identify more than four substances in a mixture [32], studies with mice [33] show that mice are capable of identifying a target substance amongst 14 other simultaneously presented components. This indicates that, while humans and other mammals smell using the same mechanisms [34], the difference in amount of sensor cells and number of genes in the olfactory repertoire between species could result in an enhanced ability to differentiate a single odour in mixtures. In this context, it should be noted that mice and rats have even more functional odour receptor genes than dogs [3].

The neurophysiological processes behind the odour masking of individual substances in odour mixtures are complex. In addition to competitive inhibition at the level of odour-binding proteins and olfactory sensory cells, inhibition of stimulus transmission at the level of the glomeruli and higher signal processing areas cannot be ruled out [35,36,37,38].

As the olfactory mechanisms of mammals are similar [34], it is possible that masking impacts the effectiveness of an explosive detector dog.

While there are different forms of masking, this paper restricts itself to masking by suppression, with the addition of a substance to cover the traces of the scent. Masking through restriction of the release of components, e.g., with packaging or absorption, is not considered.

## 2. Materials and Methods

### 2.1. Test Environment Setup

**Figure 1:**
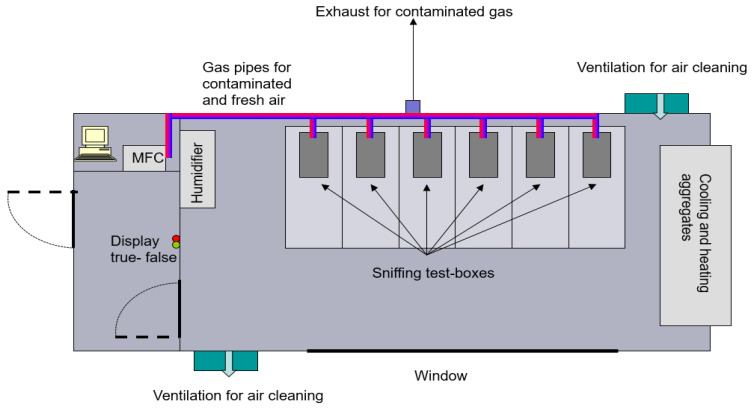
Layout of the test environment. It consisted of a 12 m × 2.44 m container. Technical equipment for heating and cooling (as well as humidifying the container chamber), a mass-flow controller unit (MFC) for contamination experiments and six sniffing test boxes containing explosive or inert odour samples were installed inside. A true–false display was mounted at the exit to indicate the result of the test run to the dog handler after the test.

Figure 1 shows a schematic drawing of the test environment. It consisted of a 12 m × 2.44 m container that was adapted from a commercial 40 ft. reefer container purchased from Braun Container Handels-GmbH, Hamburg, Germany.

The temperature inside the container could be varied between −20 and 45 °C ± 2 °C, and humidity could be adjusted from about 15 to 90% ± 10%. The complete container was inside an assembly hangar, so experiments could be performed throughout the year under any conditions of temperature and humidity. Cross ventilation enabled the rapid exchange of the air inside the container if indoor contamination should have occurred or if the environmental conditions, for example, from high to low temperatures, were required to be quickly changed.

**Figure 2:**
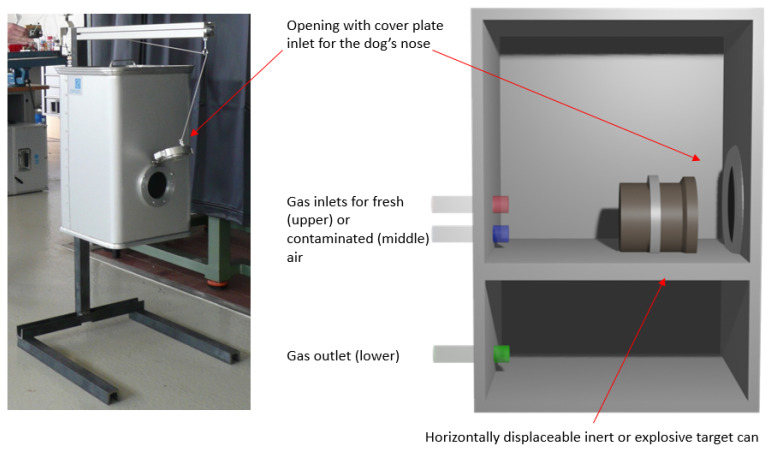
Picture and schematic of the sniffing test box. An inert or target can was placed within the test box. Sniffing dogs had to insert their nose into the opening of the test box.

Figure 2 shows an image and a sketch of an aluminium sniffing test box. Distractor or explosive odour cans could be placed behind the vertically mounted opening of the box. In this experiment, TNT target odour cans (hereafter referred to as target cans) were used. The cover plate could be opened with a cord by the operator inside the container.

Two gas inlets for fresh and contaminated air allowed for an adjustment of defined concentrations inside the box using mass-flow units. A gas outlet led the contaminated air directly outside the container.

**Figure 3:**
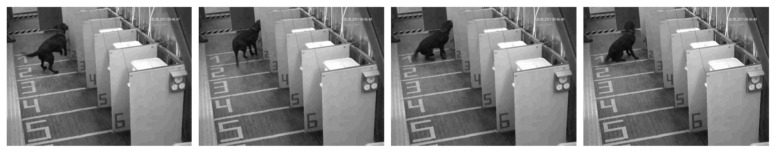
Example of the test run with a sit indication at box 1.

The dogs were trained to insert their nose into the opening. This made sure that the sniffing process proceeded within the contaminated air and near the odour sample. They were taught to scent the boxes from box one to box six and then back again when necessary until they found the target scent and indicated their detection by alerting with a trained behaviour (sitting in front of the box) as shown in Figure 3. For the experiments themselves, the search run of the dog was carried out without any interaction of the dog handler beyond the signal to start searching.

Each run contained exactly one positive target and 5 inert distractors. Two interchangeable target cans and 12 interchangeable distractor cans, which were all exchanged every two weeks, were used for the set of six test boxes. Which target can and blank can were used for each run was selected randomly. The placement of the test box containing the target can was also randomly varied. A second set of boxes was made and stored separately to allow the boxes to be exchanged after several days or in case of an observed contamination.

A chief operator controls all experiments from a separate office. Audio and video connections to this separate control station, which enabled control and interpretation of the canine search and detection process, were set up.

After setting up the temperature and humidity inside the container chamber, the chief operator could start the experiment by remotely programming the mass-flow controller. The chief operator only had access to the experiment number, which was linked in the system to the position of the target can. The staff inside the container set the correct valve positions, placed the test box containing the target can in a predetermined position and opened the cover of the boxes with a lever. After placing the boxes, all of the boxes were touched to obscure the human odour as a cue. Neither the chief operator nor the dog handlers had any information about the position of the test box with the target can inside before the first run. The chief operator monitored the search performance of the canine and entered the number of the indicated box into the system, which sent a signal to the light inside the container. This was either green, for correct, or red, for incorrect, and the chief operator did not see the rewarding or lack thereof and therefore did not know the position of the target can. The handler inside the container immediately received this information to confirm the dog. All technical data and a video stream of the experiment, as well as all dog-related results, were stored for later evaluation.

**Figure 4:**
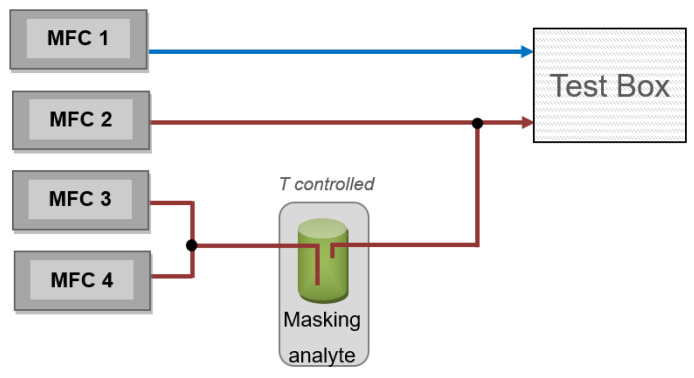
Details of the mass-flow controller unit.

Figure 4 shows the setup of the mass-flow unit. It consists of four mass-flow controllers (MFCs). MFC 1 provided a flow of fresh air to the box. MFC 2 to MFC 4 provided a defined concentration of the masking agent. By adjusting the temperature of the liquid masking substance, the gas concentration of the air stream was set according to the vapour pressure.

The staff inside the container set the valves for fresh and contaminated air according to the predefined experimental program. The MFCs, as well as the valves, were regulated in a way that every test box was supplied with the same flow of fresh or contaminated air.

### 2.2. Odour Source

**Figure 5:**
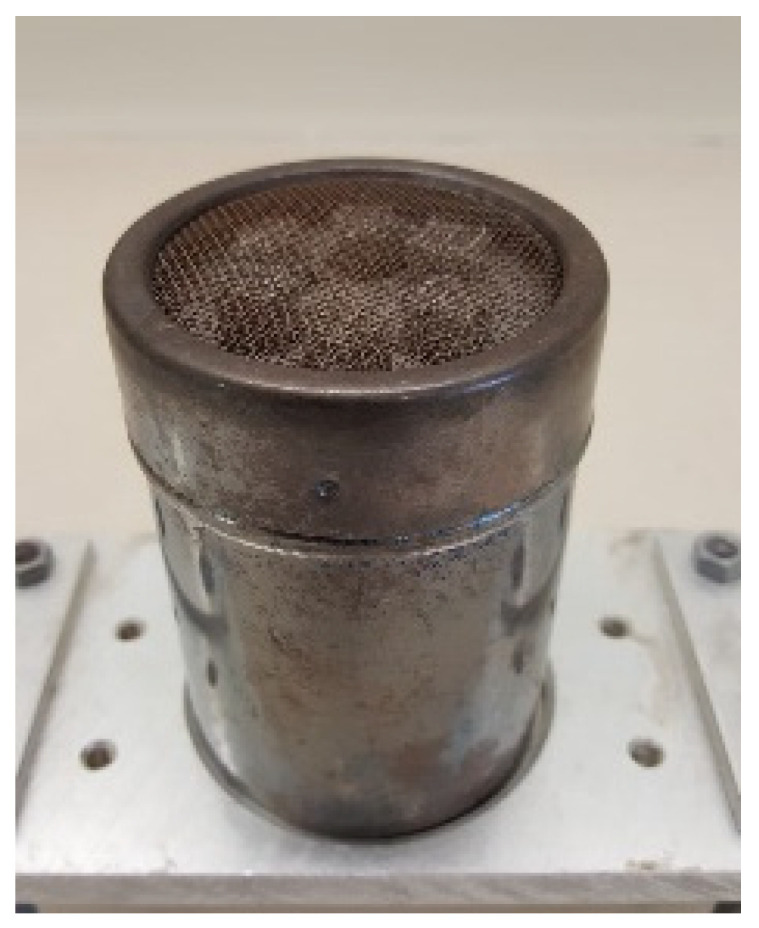
A TNT target odour can with a diameter of 7 cm. The metal can had six EMPK^®^ test bodies prepared with technical TNT inside it.

Another important aspect of these experiments was the development of a suitable target odour source. Figure 5 shows an image of the source used. It was realised as a metal can with a diameter of 7 cm containing six EMPK^®^ (Echtstoff-Mikromengen-Prüfkörper or micro-amount testing devices, ExploTech GmbH, Siegburg, Germany) test devices which exhibit a reproducible and uniform emission rate at room temperature (Figure S1) [39]. The EMPK^®^ was filled with carrier material. The carrier material could be prepared with a certain amount of any kind of explosive or as a distractor without any substance. The EMPKs were inserted into cans that were protected by a net lid to prevent direct contact with the dog’s noses. Since EMPKs contain explosives but are not classified as Class 1 dangerous goods or explosives according to ADR or German explosives law, the handling (transport, storage, use, disposal) of the samples in the context of the experiment is considerably simplified. Potential problems from a labor law and legal perspective due to the simultaneous presence of explosives and other hazards, such as the combustible substances used for masking, can also be avoided. The possibility of not storing the odour sources in the usual explosive storage facilities and the small quantities of substances reduce the risk of cross-contamination.

For the experiments two types of cans were used: distractor cans were filled with six distractor EMPKs while the target can was filled with six EMPKs prepared with 2,4,6-trinitrotoluol (TNT) (DynITEC GmbH, Troisdorf, Germany, military grade). The rate of evaporation of TNT in the target can was found to be reproducible and remained constant over a long time but depended on temperature and substance (see Supplementary Materials). Using this setup for the target can, contamination of the box and the test environment with traces of explosives was minimised, so false indications by spreading traces of explosives were less likely.

The target cans represented a strong localised odour source. From the LC-MS measurements, the strength of the source and the period of application could be calculated for various temperatures. At room temperature, the evaporation rate of this target odour can was about 0.4 µg/h. Saturation effects were not considered.

### 2.3. Masking

#### 2.3.1. Masking Substances

Petroleum and n-decane (purchased from Sigma-Aldrich, Taufkirchen, Germany) were used as masking substances. Petroleum was selected, as it was assumed to be a candidate for the masking of an IED during real operations. Based on the work of L. Waggoner, a less complex substance (n-decane) with a similar vapour pressure and structure (both hydrocarbons) was selected to investigate whether a difference between the two substances could be found. As petroleum is a mixture of different VOCS, changes in the chemical pattern in the gas phase need to be considered due to loss of highly volatile trace constituents. This was tested with thermal desorption–gas chromatography–mass spectrometry (TD GC-MS) (Figure S2). To create a stable chemical pattern in the headspace, 500 L of ambient air was bubbled through 5 L of petroleum. All used petroleum was aged in this way and exchanged after a maximum of 1 week. For masking experiments, all blank boxes and boxes containing the target odour can were subjected to petroleum or n-decane (depending on the scenario).

#### 2.3.2. Validation of the Concentration of Masking Substances in the Test Boxes

The upper and lower target concentration ranges for petroleum and n-decane are listed in Table 1. The concentrations were measured with a photoionisation detector (PID) (Minirae 3000 with a 10.6 eV lamp, Honeywell, NC, USA). Different procedures were carried out to set the concentrations, and the reliability of the procedures was proven with a validation:

- Procedure to set the high-concentration range: A beaker loosely filled with absorbent paper sheets was placed in a box. The liquid substance (V = 50 mL ± 10 mL) was poured over the paper sheets. The box was closed with a lid, and after 30 min, the first measurement was carried out.
- Procedure to set the low-concentration range: To set the lower concentration of substance in the boxes, 8.0 µL (n-decane) or 5.7 µL (petroleum) was injected with a pipette into the box while the lid was slightly lifted. After 30 min the first measurement was carried out.

When the concentration was within the required range, the procedure was continued, and when outside the required range, the procedure was stopped and repeated from the beginning. If the procedure was continued, a gas flow was started that was led through the substance and into the box. The MFCs were adjusted in a way that the chosen concentration inside the box was nearly constant. The boxes were then ready for use in the experiments. After the experiments, the boxes were cleaned. The above-described procedures for low and high concentrations were successfully validated within the range shown in Table 1.

**Table 1:** Concentration of petroleum and n-decane used for the masking experiments.

Substance and Abbreviations		Concentration Range [ppmV]
Petroleum	High conc.(P+)	350–450
	Low conc. (P−)	5–15
n-Decane	High conc. (D+)	575–725
	Low conc. (D−)	5–15

### 2.4. Selection of Dogs

Eight dogs ranging in age from 1 to 3 years were used for the tests; five females and three male dogs were selected, of which six were Labrador Retrievers, one a Golden Retriever and one a Malinois. None of the dogs had previously been deployed on real searches. All the Labrador Retrievers were trained as detector dogs prior to the study and, as such, were trained in the routine of searching for a substance. The dogs were mainly trained on TNT pellets in the range of 10–15 g. Other explosives were not trained prior to the experiments described in this paper. The Golden Retriever and the Malinois had no experience in searching. All dogs were trained by the same trainer with identical methods for the study in order to have eight similarly trained dogs. Three dog handlers took part in the training and in the study.

### 2.5. Dog Training

Dogs were trained in two steps: first familiarization with the target can and then the training of the searching sequence. The dogs were rewarded with a clicker and food as the primary reinforcer.

Dogs were scent trained using modified cans holding six TNT EMPK, which were later used in the experiments. Dogs were rewarded for intense sniffing of the cans, which were regularly replaced to avoid an over-specific association with one can. After three weeks, they could accurately indicate the presence of target cans, and the next step of training was carried out. The dogs were taught to search the test boxes at the opening of the box, moving from box one to box six and, if they did not find the target can, to search from box six to box one. They alerted by sitting when a target can was scented. Blank runs (runs without target odour) were also integrated, to ensure that dogs did not expect that a target was always present.

During the training and the experiments, the welfare of the dog was always considered, and care was taken to avoid extensive stress from longer than necessary training/testing sessions. Dogs who were having difficulties due to temporary health reasons (controlled by a veterinarian) were removed from the testing until they recovered.

### 2.6. Experimental Setup and Canine Trials

The trials were compiled of different sections: preparation of the container, dog search/experiments and cleaning.

#### 2.6.1. Preparation of the Container

The cleaned boxes were prepared (using disposable gloves) by the container staff and chief operator. The distractor cans were placed into their respective boxes first, and, finally, the target can was placed in its box. The target can was always the last to be handled, and the blank and target cans were always stored separately but in the same room in different boxes/bags received from the same batch to prevent the cans taking on different environmental odours over time. The samples were stored as recommended by L. Lazarowski et al. [40]. If the samples needed to be removed from the boxes for any reason, they were placed on separate pieces of aluminium foil, which were disposed of after replacing the cans in the boxes.

After the setup was completed and the masking concentration for the scenario was reached, all persons involved in the setup inside the container touched all boxes on the sides, where they were carried in, on the front flap and on the pull rope.

The first experimental scenario carried out was the ‘90% test’; a correct indication should be achieved at least 90% of the time for TNT-only trials. It was carried out under the exact conditions of the masking tests, minus the masking substance.

Passing the 90% tests was obligatory for starting the subsequent masking scenarios: low-concentration petroleum, high-concentration petroleum, low-concentration n-decane and then high-concentration n-decane. Occasional tests with no masking substance were carried out between the masking tests to ensure that the dogs were still performing at the same level as the initial tests for the target can without other odours.

#### 2.6.2. Dog Search

The dog and handler entered the container. At this point, the dog handler and chief operator did not know in which box the target can was hidden.

In order to keep the concentration of the masking substance the same throughout the runs, the cover plate (shown in Figure 2) was kept closed until shortly before each run and was closed quickly after the dog finished its search. After the cover plate was opened, the dog was instructed to search the boxes independently until it indicated at a box or gave no indication. The chief operator entered the number of the alerted box that he observed through the video stream into the software, which then displayed a green or red light in the container depending on whether the alert was correct or incorrect, respectively. The dog handler subsequently rewarded the dog for a correct indication or silently led the dog out of the container in the case of an incorrect response. To prevent the body language of the dog handler influencing the dog, the handlers were instructed to turn their back to the dog until the light signal was given.

The dogs were allowed to check boxes up to 2 times. If this was exceeded, the dog was recalled, and the handler silently left the container. The test was marked as ‘not found’ and was considered an incorrect result (false negative).

After the dog and handler left the container, the next dog was sent in until the experiment was complete. After cleaning and rearranging the boxes, the next experiment began.

If the alert of one dog was a false positive, the dog was not rewarded and was led out of the container, and the next run began. Subsequent false positives on the same box caused a discontinuation of the tests. The box was tested onsite with an Itemiser 3DX (ELP GmbH, Germany). Using an already established method [41], methanol/water (50/50) wetted swabs were taken, solvent was extracted, and the extract was analysed by LC-MS^3^. Boxes were thoroughly cleaned and checked for remaining TNT contamination. Experience showed that repeated false positives on the same box always led to confirmed contamination. No repeated false positives occurred when there was no contamination.

The first scenario was the 90% trial (TNT-only); these were carried out over nine days. Nine days of tests with a low concentration of petroleum were then carried out, with two TNT-only experiments on two mornings. A high concentration of petroleum was tested over eight days, with four TNT-only experiments (two experiments on two separate mornings). After 1 day of TNT-only experiments, 6 days of a low concentration of n-decane were directly followed by 7 days of a high concentration of n-decane.

#### 2.6.3. Cleaning

After each experiment (all participating dogs complete one run), all test boxes and the sieves on the cans were cleaned with isopropanol and paper towels to prevent contamination (for example, through saliva transfer). The ground of the container was also mopped, and fresh air was passed through the container for approximately 10–15 min using the ventilation for air cleaning (see Figure 1).

After the day’s experiments (between 4 and 8 experiments per day, 4–5 days a week) were completed, all surfaces of the test boxes were cleaned with isopropanol and heated with a hot air gun. The sieves on the cans were also cleaned with isopropanol (distractor first, the target can last). All cans were then stored in separate plastic bags (distractor cans together and target cans together).

Two target cans and 12 blank cans comprised one set. The ‘sets’ were exchanged throughout 2 weeks, and after the two weeks, a freshly prepared set of cans was used.

After each change of scenario (TNT-only, D−, D+, etc.), contamination of the setup was checked using swap sampling and LC-MS^3^ as already described. The cleaning procedure was carried out until the LC-MS^3^ results were negative.

### 2.7. Statistical Analysis

To analyse whether the results of the masking trials differed from those of the 90% trials, a Fisher’s exact test was carried out.

Fisher’s test is considered passed when the *p*-value is larger than 0.05. A *p*-value larger than 0.01 but smaller than 0.05 indicates the possibility of a difference; however, a significant difference cannot be safely concluded [42].

It was felt by the dog handlers that the dogs spent more time searching with high concentrations of a masking substance in comparison to only the target can. To inspect this, the time taken until alert was recorded from the moment of release until a clear signal was given. Runs where it was unclear exactly when the dog alerted or when technical problems occurred were not included.

To determine whether there was a difference in the alert time for different scenarios, a *t*-test was carried out. The analysis of *p*-values is analogous to that of Fisher’s exact test. The *t*-test is considered robust to distribution variations when, as in our data, both samples are equally distributed. As the data, however, is strongly right-tailed, the logarithm of the time was taken to create more normally distributed data, as suggested by Samuels and Witmer [42].

### 2.8. Additional Considerations

The experiments were carried out without the regular use of blank runs and without the use of varying distracters. The authors are aware that this is unusual when carrying out scent trials with dogs. The authors decided against the use of blank runs/varying distracters to remove as many external influencing factors as possible. The goal of the trials was to determine whether small or large amounts of two specific masking substances were able to prevent the dogs from finding the one specific scent that they were conditioned to alert to. It was thought that reducing the factors involved would result in a more meaningful comparison.

As the dog is a biological detector, there are parameters that could potentially influence the dog that do not occur in instrumental devices. Some steps taken to prevent internal or external influences are discussed below.

Preceding each test day, non-evaluated test runs were carried out, similar to running a blank sample before measuring with analytical instruments. Problems such as contamination of TNT in a theoretically clean box or performance/motivation of the dog were therefore detected early, and solutions could be found. Dogs with expected atypical restrictions of their performance (e.g., on heat, previous veterinary appointments, etc.) were excluded from the assessment for that day but were allowed to perform test runs. Runs of these dogs were marked as ‘do not evaluate’.

To prevent a drop in the results of the dogs due to habitation and boredom, varying searches (different substances, distractions, no target substances) were also carried out at irregular intervals both inside and outside the container. These runs were not included in any analysis.

For the statistical analysis it must be considered that there were very few errors to base the analysis on. Additionally, it is not known which factors affected a correct result.

## 3. Results

For the evaluation of the results, sensitivity was calculated. In conformity with the norm, selectivity is also calculated; however, as the experiment was not carried out in anticipation of calculating true negatives, the selectivity does not have a very high informative value and has therefore only been included in the Supplementary Materials. Attempts marked as ‘do not evaluate’ (see Section 2.8) by the operator were not included in the final results.

In total, 1253 runs were carried out. Of these, 44 (3.5%) were excluded due to contamination. As 37 (3.1%) trials were discarded as per the procedure explained in Section 2.8, this left 1172 runs to be evaluated.

### 3.1. Trial Results

Three of the masking trials had a sensitivity of 99%, and one trial (low concentration of n-decane) had a sensitivity of 100%. The sensitivity for TNT-only trials was 98%. Table 2 shows the sensitivity for each of the different experiment types.

Table 3 shows the results of Fisher’s exact test.

### 3.2. Influencing Factors

Potentially influencing factors were analysed:

- Did the individual dog handler affect the error rate? Are the dogs of one dog handler better than those of another?
- Did the box position influence the error rate? Consideration of bias selection of dogs and the short-term cumulative exposure to chemicals on the dog’s nose.
- Did the error rate differ between the beginning and end of a week? Consideration of long-term cumulative exposure to chemicals on the dog’s nose.

No significant statistical variances could be found for the above mentioned influencing factors.

**Figure 6:**
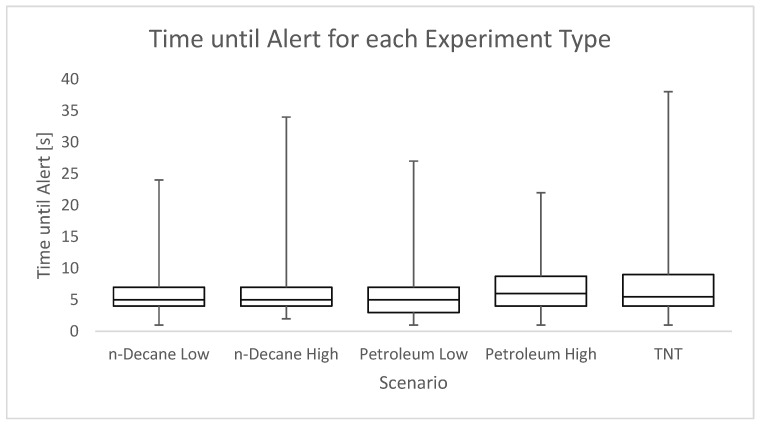
Time until alerts [s] for each experiment type. Time was taken using the timestamp on the camera from the start of the dog’s search to the first indication.

Table 4 shows the results of the *t*-test for the comparison of low and high concentrations of both petroleum and n-decane. Figure 6 shows the box and whisker plot for the times for each separate scenario. For individual alert times for each substance, see.

## 4. Discussion

During the experiments, contamination sources were detected, and the methods of the experiment were refined. Using the final refined method described in this paper, contamination could be kept to a minimum.

The documented results show a very high performance of all eight dogs, with the lowest sensitivity for any masking substance being 99%. Fisher’s exact test showed no discernible difference in the detection rate of the target can in the described scenarios when masked by high and low concentrations of petroleum and n-decane.

This does not support the previous finding of Laing [30] and Waggoner [31], who showed that masking with a high concentration of a substance was possible. One possible reason could be the different substances used in the experiments; different substances may prove to be more effective at masking than others.

In general, the results shown are better than expected. This could be due to several different factors. As mentioned in Section 2.8, no varying distractors and blank runs were incorporated into the experiments. This results in a test for the dogs that is significantly easier than tests where there are blank runs and/or varying distractors in addition to the correct answer. The dogs went through a very long training phase for the used materials, and six of the eight dogs were trained from puppies to be scent detection dogs. The non-evaluated test run each morning (comparable with a blank run of an analytical instrument before analysis to ensure the device is working correctly) could also increase the accuracy of the results, as dogs who, as living beings, have an ‘off’ day were detected and removed. This was possible as the aim of this experiment was not to determine how well each dog performs on an everyday basis but rather when the dog is performing at peak capacity to determine whether there is biological masking caused by the two selected substances. The results did not show any correlation between any potential influencing factors and correct results. However, because of the complex nature of dogs as scent detectors, it cannot be ruled out that there is an unknown influencing factor resulting in the high results. To the authors’ best knowledge, the results were not influenced by any external factors.

The short- and long-term tests show no difference from each other. No correlation could be found between error rates and box position, which indicates that there were no short-term effects on the mucosa, nor were there differences between the first and last tests within one week, showing that there was no long-term effect on the olfactory mucosa.

There was a significant difference in time to alert between n-decane and petroleum. According to M. Fletcher et.al. and N Mandairon et. al., a rat’s perception of scents can improve over time [43,44,45]. As petroleum was the first substance to be tested, it is possible that the dog’s ability to detect the target can could have improved over the course of the scenarios, resulting in the later scenarios (n-decane) being detected faster. It can also not be ruled out that the difference could be due to the effect of the complex composition as theorised by Waggoner in his experiments with two different masking substances.

However, this is not believed to affect the results for a few reasons. Firstly, no difference between the results of blind dogs and all dogs can be seen. Secondly, due to unexpected contamination, some dogs gave an incorrect response (this was subsequently swabbed, and a concentration in a lower µg range was determined) at particular boxes. This contamination was known to no one, and despite receiving no reward and searching again, they were very confident in their alert and showed repeated alerts at the contaminated sites. This indicates that the dogs truly detected the target can and were not simply ‘reading the room’. This also indicates that they were not simply alerting to the ‘different’ cans due to different storage, etc. This is also rather unlikely, as care was taken to store all samples separately in identical boxes from the same batch.

By using a target can instead of mixing technical TNT vapour with the masking substances in the gas phase, there was not a constant concentration of technical TNT in the gas phase. Instead, there was an increasingly strong odour plume the closer to the source one was. This was closer to a realistic scenario, as during a search, the target substance also emits from a source and not solely in its vapour form. As extensive testing of both the target and masking substances was carried out before the experiments, it is unlikely that the concentration of the masking substance or target substance deviates to such a high level as to affect the experiments.

## 5. Conclusions

The designed testbed allows the scientifically reliable testing of canine detection capabilities. Through the control of temperature and humidity within the testbed, environmental factors could be minimised, allowing the tests to focus on the masking of explosives. The masking substances had a controlled and defined concentration, which led to a reproducible test environment. The testbed could be used to test the effects of the masking of explosives using suppression on the effectiveness of canine detection abilities.

In total, 1172 runs were evaluated, of which only 12 were false positives and 4 false negatives; the masking of the substance emitting out of an explosive target odour can by suppression using two different substances (petroleum and n-decane) in high and low concentrations was not successful. No influencing factors such as running order, day of week and box position could be found. The only difference could be found in the time to alert between n-decane and petroleum, which could be due to either a learning effect of the dogs or a complex composition.

Our experiment was based on dogs who, for the most part, have experience in searching for substances and who achieved a high standard of detection without any interfering elements. Based on our experiments, the two selected masking substances cannot mask our target in a controlled situation. In real scenarios, this could be very different due to the many uncontrolled interferences and conditions. More experiments with different substances are necessary to determine whether other substances could be effective in masking.

## Figures and Tables

**Table 2:** Sensitivity for TNT-only trials and each masking trial.

Experiment	Sensitivity [%]
90% Trials (TNT-only)	98
n-Decane Low	100
n-Decane High	99
Petroleum Low	99
Petroleum High	99

**Table 3:** *p*-values for the comparison of TNT-only trials with each subsequent masking trial. The *p*-Value is the *p*-value calculated from Fisher’s exact test.

Experiment Type	*p*-Value
n-Decane Low	0.162
n-Decane High	0.423
Petroleum Low	1.000
Petroleum High	1.000

**Table 4:** Results of the *t*-test for the comparison between low and high concentrations of n-decane and petroleum. The *p*-value represents the *p*-value for the corresponding χ^2^ statistic and respective DoF for each test. Significance presents whether a difference is discernible from the results. An ‘x’ indicates that no significance can be seen at α = 0.01 or α = 0.05 from the results for this sample, while ‘**’ indicates that the results for this sample can be considered significant at α = 0.01.

*t*-Test Comparison of Time Until Alert			
	*p*-Value	DoF	Significance
n-Decane	0.642	379	x
Petroleum	0.0000300	401	**

## Data Availability

The original contributions presented in this study are included in the article/Supplementary Materials. Further inquiries can be directed to the corresponding authors.

## Supplementary Materials

The following supporting information can be downloaded at https://www.mdpi.com/article/10.3390/ani16040656/s1, Figure S1: Amount of TNT (N = 3) sampled via Tenax TA filled sampling tubes (@50 mL/min) from TNT target cans at different temperatures and sampling times, as well as a scheme of the test setup used. The Tenax tubes were then extracted with acetonitrile and analysed using LC-MS^3^; Figure S2: GC-MS chromatograms of the headspace above the petroleum used, collected using a Tenax TA sampling tube (from top to bottom: fresh, after 300 and 616 h of aging). Initially present, more volatile components largely disappeared after aging.

## Abbreviations

The following abbreviations are used in this manuscript:

EMPK	Echtstoff-Mikromengen-Prüfkörper or micro-amount testing devices
TD GC-MS	Thermal desorption–gas chromatography–mass spectrometry
IED	Improvised explosive device
LC-MS^3^	High-performance liquid chromatography–triple quadrupole mass spectrometry
MFC	Mass-flow controller
PID	Photo ionisation detector
TNT	2,4,6-Trinitrotoluene
