# Supplementary material for: Investigation of the Impact of Extraneous Odours on the Detection Capability of Explosive Detection Dogs Under a Controlled Test Environment
Source: Animals (Basel). 2026 Feb 19;16(4):656. doi: 10.3390/ani16040656 (PMC12937438; doi:10.3390/ani16040656)
Supplement: Supplementary file 1 [file animals-16-00656-s001.zip › animals-4135968-supplementary.pdf]

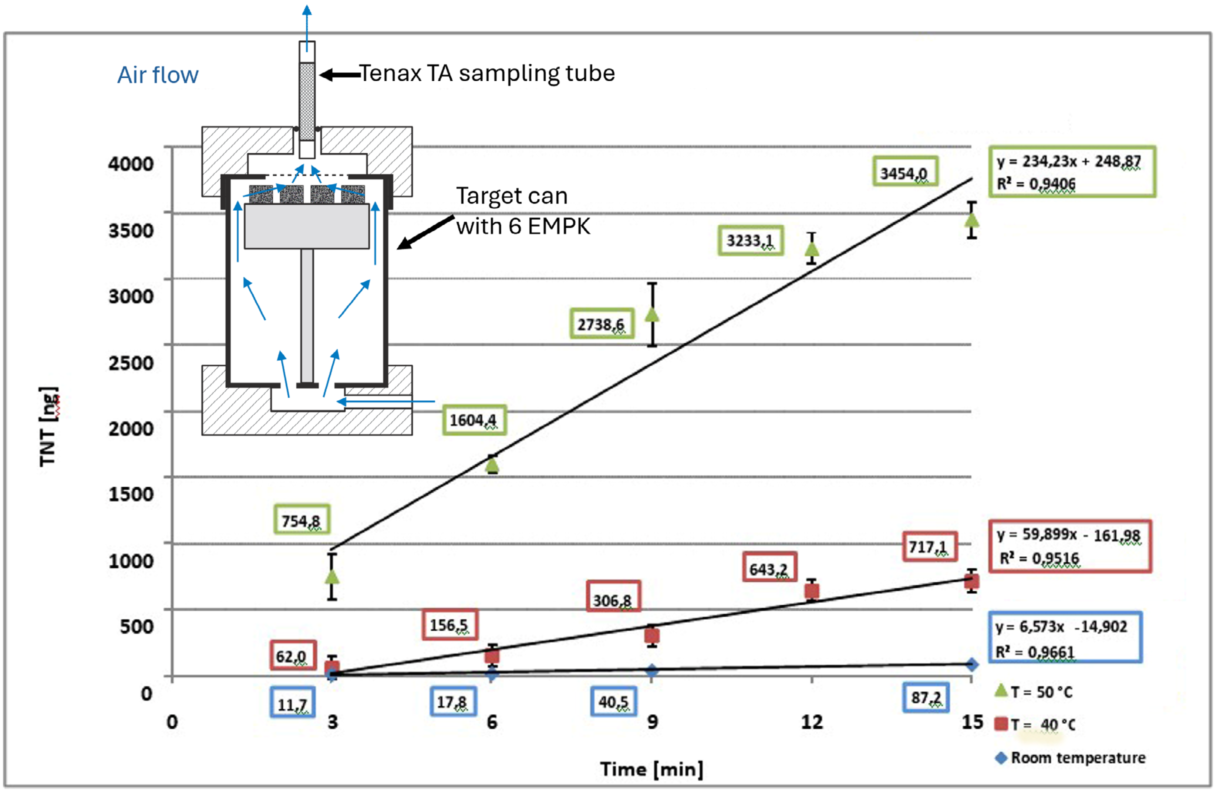

Figure S1: Amount of TNT (N = 3) sampled via Tenax TA filled sampling tubes (@50 ml/min) from TNT target cans at different temperatures and sampling times, as well as a scheme of the test setup used. The Tenax tubes were then extracted with acetonitrile and analyzed using LC-MS[3].

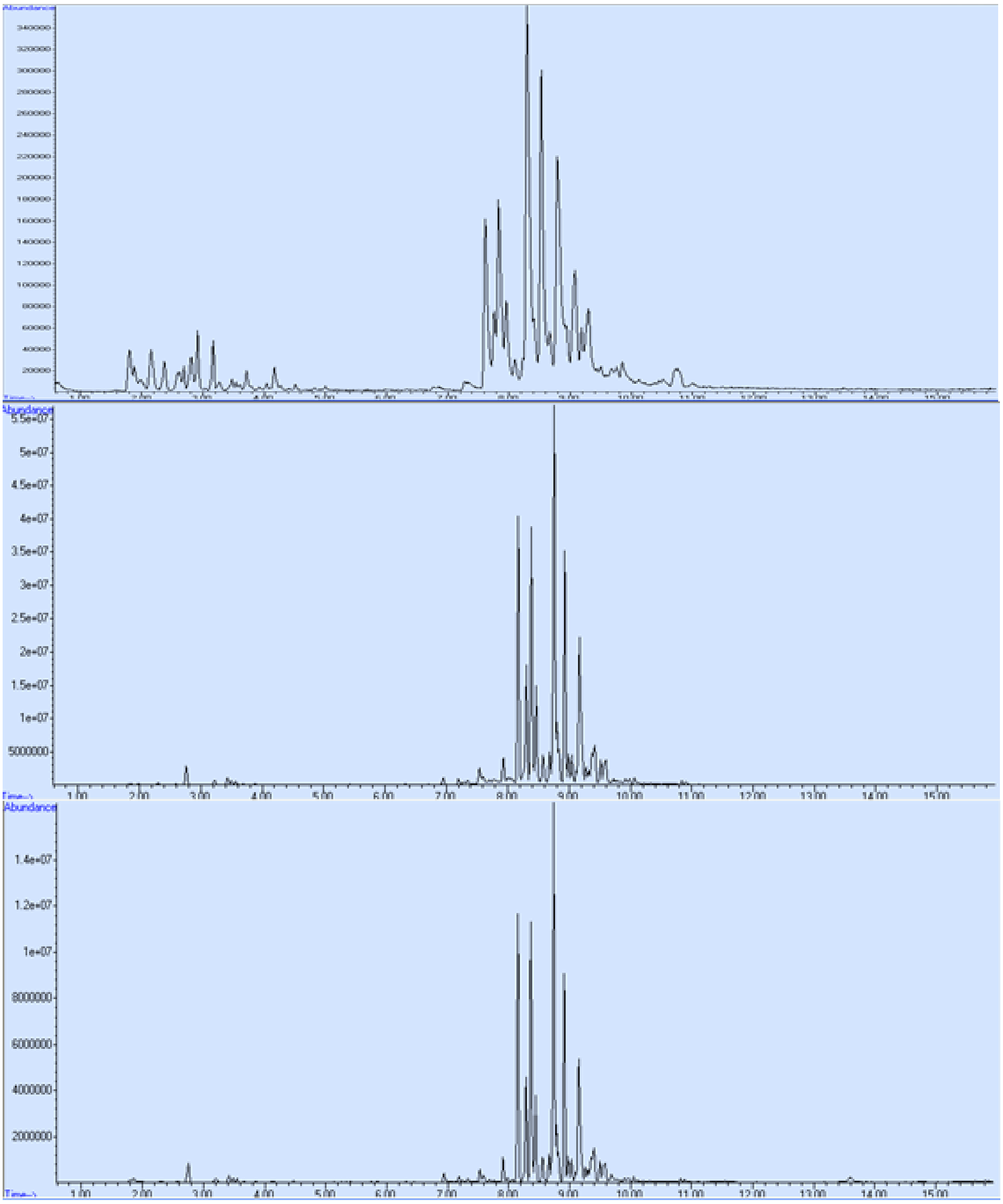

Figure S2: GC-MS chromatograms of the headspace above the petroleum used, collected using a Tenax TA sampling tube (from top to bottom: fresh, after 300 and 616 hours of aging). Initially present, more volatile components largely disappeared after aging.
